# PlgFormer: parallel extraction of local-global features for AD diagnosis on sMRI using a unified CNN-transformer architecture

**DOI:** 10.3389/fneur.2025.1626922

**Published:** 2025-08-29

**Authors:** Guoxin Wang, Yuxia Li, Zhiyi Zhou, Shan An, Xuyang Cao, Yuxin Jin, Zhengqin Sun, Guanqun Chen, Mingkai Zhang, Zhixiong Li, Feng Yu

**Affiliations:** ^1^College of Biomedical Engineering and Instrument Science, Zhejiang University, Hangzhou, China; ^2^Tangshan Central Hospital, Tangshan, Hebei, China; ^3^Electrical and Information Engineering, Tianjin University, Tianjin, China; ^4^School of Electrical and Information Engineering, Tianjin University, Tianjin, China; ^5^JD Health International Inc., Beijing, China; ^6^Department of Neurology, Beijing Chao-Yang Hospital, Capital Medical University, Beijing, China; ^7^Department of Neurology, XuanWu Hospital of Capital Medical University, Beijing, China; ^8^Karamay Integrated Traditional Chinese and Western Medicine Hospital (People's Hospital of Karamay), Karamay, China

**Keywords:** Alzheimer's disease diagnosis, attention mechanism, computer-aided diagnosis, sMRI, multi-level feature fusion

## Abstract

**Introduction:**

Structural magnetic resonance imaging (sMRI) is an important tool for the early diagnosis of Alzheimer's disease (AD). Previous methods based on voxel, region of interests (ROIs) or patch have limitations in characterizing discriminative features in sMRI for AD as they can only focus on specific local or global features.

**Methods:**

We propose a computer-aided AD diagnosis method based on sMRI, named PlgFormer, which considers the extraction of both local and global features. By using a combination of convolution and self-attention, we can extract context features at both local and global levels. In the decision-making layer of the model, we design a feature fusion module that adaptively selects context features through a gating mechanism. Additionally, to account for changes in image input resolution during the downsampling operation, we embed a dynamic embedding block at each stage of the network, which can adaptively adjust the weights of the inputs with different resolutions.

**Results:**

We evaluated the performance of our method on dichotomous AD vs. normal control (NC) and mild cognitive impairment (MCI) vs. NC, as well as trichotomous AD vs. MCI vs. NC classification tasks, using publicly available ADNI and XWNI datasets that we collected. On the ADNI dataset, the proposed method achieves classification accuracies of 0.9431 for AD vs. NC, 0.8216 for MCI vs. CN, and 0.6228 for the AD vs. MCI vs. CN task. On the XWNI dataset, the corresponding accuracies are 0.9307, 0.8600, and 0.8672, respectively. The experimental results demonstrate the high precision and robustness of our method in diagnosing people with different stages of cognitive impairment.

**Conclusion:**

The findings in our experimental results underscore the clinical potential of our proposed PlgFormer as a reliable and interpretable framework for supporting early and accurate diagnosis of AD using sMRI.

## 1 Introduction

Alzheimer's disease (AD) is a neurodegenerative disorder that progressively impairs cognitive functions such as memory, thinking, and behavior. It is the most common cause of dementia and affects the normal lives of over 30 million people worldwide (1), primarily older adults. As the disease advances, individuals may experience difficulties with language, disorientation, mood changes, and eventually lose the ability to perform daily activities independently. These challenges not only degrade the quality of life but also place a heavy burden on families and healthcare systems. Early and accurate diagnosis of AD is therefore essential for effective intervention and management.

Diagnosis AD is based primarily on clinical evaluation, including medical history, physical examination, and cognitive tests (2, 3). Unfortunately, effective treatments for AD have yet to be discovered. Therefore, early diagnosis is imperative not only for improving the quality of life for patients but also for supporting the development of more effective treatment methods and intervention measures.

Structural magnetic resonance imaging (sMRI) is a noninvasive medical imaging technique that captures the physical structure of the brain and provides detailed images of its tissues, including gray and white matter. Unlike functional magnetic resonance imaging (fMRI), which measures changes in blood flow and neural activity, sMRI uses strong magnetic fields and radio waves. sMRI has become a valuable tool in diagnosing AD due to its ability to detect changes in brain structure associated with the disease (4–7), such as shrinkage in the hippocampus and other areas of the brain. However, its use is often limited by physician experience and time-consuming processes.

To solve this dilemma, hopes have gradually been placed on computer-aided diagnosis of AD, which has a long history and has achieved good performance. Existing methods for computer-aided diagnosis of AD using sMRI can be broadly classified into three categories: (1) Voxel-based methods; (2) Regions of interest (ROIs)-based methods; and (3) Patch-based methods. However, these methods face challenges due to the high dimensionality of sMRI data and the discrete distribution of AD lesions.

Voxel-based methods for computer-aided diagnosis of AD utilize the entire sMRI as input and extract global features for AD diagnosis. Hinrichs et al. (8) used gray matter density to extract discriminative features and employed a linear programming boosting method to classify AD and normal control (NC). Kao et al. (9) detected white matter changes throughout the whole sMRI to diagnose AD. Vounou et al. (10) detected markers associated with longitudinal changes in brain voxels caused by AD and used a sparse reduced-rank regression model to classify them. However, these methods extract features across the whole sMRI, resulting in high computational complexity and overfitting of the model due to limited data.

In contrast, ROI-based methods focus on extracting features from pre-segmented regions with lower feature dimensionality compared to voxel-based methods. Zhang et al. (11) adaptively extracted 93 ROIs using the atlas warping algorithm and identified AD with support vector machines (SVM). Liu et al. (12) proposed an ensemble classification model to construct gray matter density features within regions by using multiple spatially normalized templates. However, ROI selection is heavily dependent on specialist knowledge, making it difficult for developers to master. Additionally, ROI-based methods can only capture local discriminative features, making it difficult to capture global features such as ventricular volume and gyral sulcus morphological changes, which is insufficient for computer-aided AD diagnosis.

Patch-based methods extract features with intermediate feature dimensions, focusing more effectively on local discriminative features. Qiu et al. (13) trained a FCN to adaptively and randomly select meaningful patches, which were fed to a multilayer perceptron (MLP) for individual-level AD diagnosis. Zhang et al. (14) selected discriminative patches by computing shapley values and extracted local-global context features in sMRI using Convolutional Neural Network (CNN). However, how to combine local patch features with global representations is still a problem that needs to be explored.

**Figure 1:**
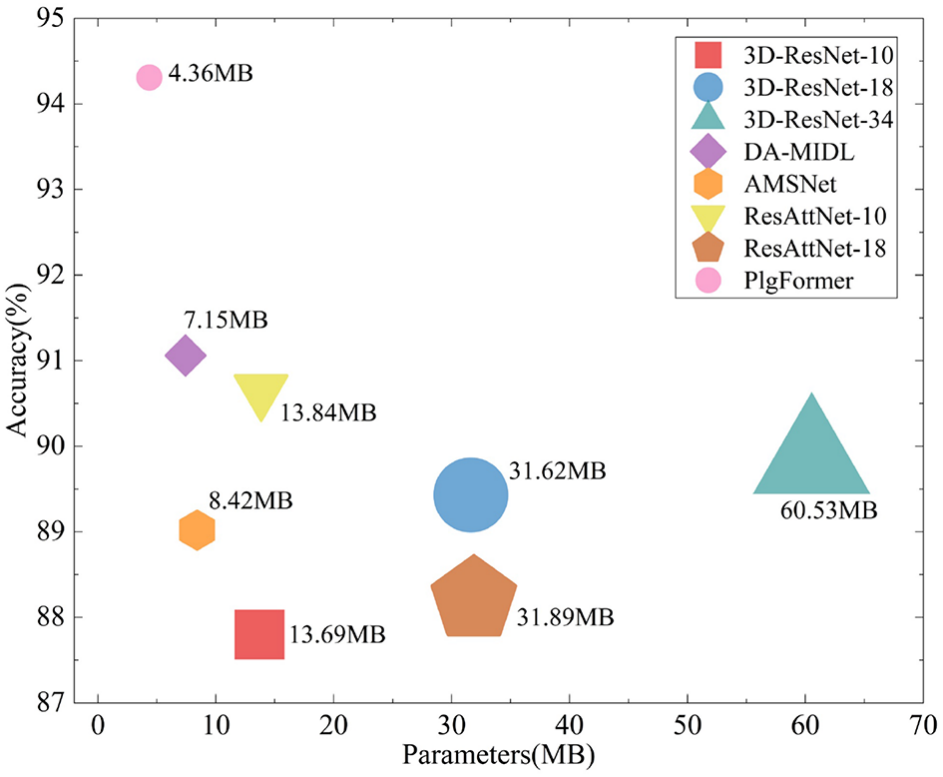
Various existing models were evaluated on ADNI dataset for the binary classification of AD and CN, and their performance was compared in relation to the number of model parameters. The graphical area corresponded to the number of model parameters. Our PlgFormer achieved optimal classification performance with the lightest number of parameters. The classification performance is illustrated in Table 1.

We propose PlgFormer, a new method for AD diagnosis using sMRI. PlgFormer utilizes convolution modules and transformer modules in parallel to extract discriminative local-global context features in a unified manner. To adaptively adjust parameter size for inputs of different sizes, we embed dynamic convolutional layers in early stages of the model. This operation also reduces the number of parameters 72 compared to conventional convolution, as shown in Figure 1.

In summary, the major contributions of this paper can be summarized as follows.

1. We design a novel dynamic embedding block (DEB) in our model, which is a combination of traditional convolution and dynamic convolution. Compared to traditional convolution, introducing dynamic convolution can help the model adaptively adjust the size of parameters based on the size of input, which reduces the model parameters while enhancing its robustness.
2. A dual-branch structure extracts local-global context features in sMRI in parallel. Considering feature alignment, we design both dual-branch operations as a multi-head architecture. Additionally, we introduce a feature fusion module (F2M) that adaptively selects local or global features at the decision-making layer of the model.
3. We collected a large-scale sMRI dataset called the XWNI dataset. Our extensive experiments, which included both the publicly available ADNI dataset and the collected XWNI dataset, show that our proposed PlgFormer substantially outperforms the existing baseline models while utilizing minimal parameters.

## 2 Related works

In this section, we provide a summary of current methods for computer-aided diagnosis of AD using sMRI data.

### 2.1 Traditional machine learning for AD diagnosis

Early methods for computer-aided diagnosis of AD using sMRI data mainly focused on extracting features and using traditional machine learning methods to analyze and classify them. Ashburner and Friston (15) compared differences in brain structure between individuals or groups to identify brain regions associated with specific diseases or cognitive functions. Klöppel et al. (16) transformed brain MRI into statistical features and employed SVM to identify early structural changes in the brain of AD patients. Similarly, Fan et al. (17) split MRI into specified regions and selected the most discriminative regions for AD diagnosis based on the statistical characteristics of each region and the inter-relationship between them, and performed the classification using SVM. Hinrichs et al. (8) randomly augmented the raw ADNI data and adopted a linear programming boosting method for classification, achieving more robust results. sMRI data are always over dimensional. Cao et al. (18) proposed a multi-kernel-based method combined with marginal fisher analysis to reduce the feature dimensionality of MRI and establish a complex mapping relationship from image to disease. Zhang et al. (11) used principal component analysis (PCA) for feature extraction and dimensionality reduction and conducted classification of AD and CN instances using SVM. Abuhmed et al. (19) proposed two novel hybrid deep learning models, DFBL and MRBL, which integrate multivariate BiLSTM architecture with traditional machine learning models to enhance the prediction of Alzheimer's disease progression using multimodal time-series data.

However, these methods mentioned above present two limitations: (1) manual feature extraction is reliant on human experience and may ignore discriminative features; (2) high-dimensional features may lead to overfitting.

### 2.2 CNN-based methods for AD diagnosis

CNNs have achieved considerable amount of success in the field of computer vision, with their strong inductive bias performs well in tasks including image classification (20–22), object detection, semantic segmentation, and video understanding. Naturally, these CNN-based methods were applied to AD diagnosis using MRI data, achieving satisfactory performances. Lian et al. (23) proposed a hierarchical full convolutional network (H-FCN) to automatically identify discriminative local patches associated with AD in the whole brain sMRI. Zhu et al. (24) proposed a dual attention multi-instance deep learning network to extract discriminative features from local patches and aggregate these features by attention-aware weights. Wu et al. (25) proposed a 3D CNN model to extract and integrate robust multiscale spatial features to promote AD computer-aided diagnosis. Zhang et al. (26) proposed a residual self-attention deep neural network to capture local spatial features in sMRI, while attention mechanisms are still necessary to jointly construct global representations. A multi-branch convolutional network was proposed in (27), where each branch extracts its own features independently and uses a fully connected layer for feature aggregation to obtain global representations. Zhang et al. (28) proposed a multi-relation reasoning network (MRN) that constructs brain graphs from sMRI data to capture spatial and topological relationships, improving Alzheimer's disease diagnosis through enhanced feature representation and global reasoning.

In addition to specialized CNN-based models, general models such as VGGNet (21) and ResNet (22) have also been applied to AD diagnosis due to their successful performance in natural visual scene analysis. Billones et al. (29) utilized a modified VGGNet model for AD diagnosis using sMRI data. Li et al. (30) employed the ResNet model to the ADNI dataset, focusing specifically on local discriminative features in hippocampal regions. These methods all use the strong inductive bias of CNNs to extend the conventional 2D convolutional kernels to 3D and extract the local representations associated with AD in sMRI.

The strong inductive bias of CNNs enables them to capture local features effectively even with a limited number of samples. However, when constructing global representations by integrating local information, attention mechanisms are typically still required.

### 2.3 Attention mechanism-based methods for AD diagnosis

Attention mechanisms excel at uniting different local features to construct global representations. Thanks to this, Wu et al. (25) aggregated the local features extracted by the convolution module under the supervision of the attention weights. similarly, Zhang et al. (26) directed the network to focus on critical information in the CNN feature maps and suppress non-essential features by using self-attention scores. Liu et al. (31) aggregated the semantic features between different channels by the classical Squeeze and Excitation (SE) module. Zhang et al. (32) embedded a connection-wise attention mechanism in DesNet for associating context features of the model. Jin et al. (33) constructed a simple convolutional layer for computing attention weights to retrace global features for local features extracted by convolution. Transformer is a pure attention mechanism architecture, originally proposed for natural language processing tasks (34) and has since been extended to the field of computer vision (35, 36) demonstrating progressively satisfactory performance.

However, Jang and Hwang (37) have experimentally demonstrated that the coarse embedding of Transformer into a mature CNN architecture may not always yield satisfactory performances due to its lack of focus on local features, implying that attention may not be all need for AD diagnosis with sMRI data.

## 3 Method

In this section, we present the details of the proposed method. The overview of the method is elaborated in Section 3.1. After that, dynamic embedding block, convolution and self-attention parallel extraction of local-global context features and the F2M are described in Section 3.2, Section 2.3, and Section 3.4, respectively.

### 3.1 Overview of the proposed method

The strong inductive bias of the convolution is beneficial for local features extraction, while the self-attention is significant for the representation of global features. Both local and global features are important for AD diagnosis from sMRI, which means we cannot simply conduct convolution or self-attention operations. To achieve this, we propose an architecture that extracts local-global context features in parallel with a uniform multi-head self-attention mechanism. Our designed F2M in the decision-making layer of the network selects discriminative local-global context features through a gating mechanism. Additionally, since downsampling changes the input resolution at each stage, we embed a dynamic embedding block in each stage of the network to adjust the weights for different input resolutions adaptively.

**Figure 2:**
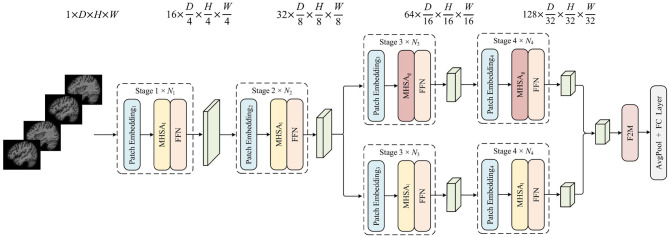
Overview of the proposed PlgFormer. This method comprises four stages of feature extraction. Each stage employs dynamic convolutional style to encode location information, MHSA_*l*_ or MHSA_*g*_ to extract local or global features, and a feature fusion module to combine these features and produce classification results. For further elaboration, please refer to Section 3. The figure schematically illustrates the data dimensions as a binary classification of AD and CN.

Figure 2 provides an overview of our proposed PlgFormer algorithm. Simply described, we take a vanilla 3-D sMRI *X*∈ℝ^*D* × *H* × *W*^ as an example. The training process of PlgFormer is depicted in Algorithm 1. The proposed PlgFormer consists of multi-stages, each of which conducts a downsampling operation to reduce the dimensionality of the data. To adapt to the change of input size, DEBs are introduced in each stage of the model to adaptively adjust the weights of convolution kernels for various resolutions. In the early stages of the model, Multi-Head-Self-Attention (MHSA) was introduced in a purely convolutional operation paired with multi-head style, without considering the computationally burdensome self-attention mechanism due to the large input size. Local and global discriminative features are then extracted using the convolution and self-attentive modules. To facilitate feature alignment before fusion, the convolution operation is designed as a multi-head structure unified with the self-attention mechanism. Local-global context features are fused with F2M and passed through global average pooling and full connectivity to obtain the one-hot tensor at the decision-making layer of the network.

**Algorithm 1:** The training procedure of PlgFormer.

**Input:** input images *X*∈ℝ^*D* × *H* × *W*^
**Output:** outputs Ŷ∈ℝ^*n*^ (n: number of classes)
1: **repeat**
2: Let stage *k* = 1, *loss* = 0.0
3: *H*^0^←DEB(*X*)
4: **for** *k* = 1 → *m* **do** (*m*:number of stages)
5: **if** *k* < = *s* **then** (*s*:number of shallow stages)
6: *H*^*k*^←MHSA^*k*^(*H*^*k*−1^)
7: **else**
8: $H_{l}^{k}\leftarrow {\text{MHSA}}_{l}^{k}\left(H^{s},\text{if}k-1==s\text{else}H_{l}^{k-1}\right)$
9: $H_{g}^{k}\leftarrow {\text{MHSA}}_{g}^{k}\left(H^{s},\text{if}k-1==s\text{else}H_{g}^{k-1}\right)$
10: *k* = *k*+1
11: **end for**
12: $Z_{aug}\leftarrow \text{F2M}\left(H_{l}^{m},H_{g}^{m}\right)$
13: Ŷ←AvgPool + FC(*Z*_*aug*_)
14: *loss* = Loss_function(*Y*, Ŷ)
15: θ←−∇_θ_(*loss*)
16: **until** convergence
17: **return** Ŷ∈ℝ^*n*^

The key factors of our proposed method for effective AD diagnosis include: (1) how to encode dynamic positions for inputs of different resolutions; (2) how to extract local and global features in a uniform manner using convolution and self-attention operations for subsequent feature alignment; and (3) how to effectively fuse local-global context features. These solutions will be elaborated in Sections 3.2–3.4.

### 3.2 Dynamic embedding block

**Figure 3:**
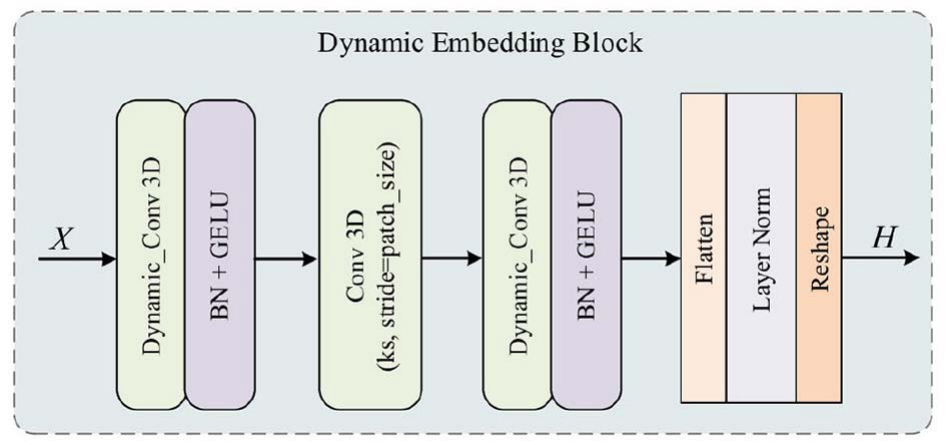
Detailed structure of the proposed DEB. The kernel size of 3D dynamic convolution is 1, while the normal 3D convolution with patch size as the kernel size and stride is used to downsample at a specific ratio. This structure enables the block to be more adaptive and reduces the number of parameters as the input image size decreases.

Dynamic convolution can adjust the size of the convolution kernel according to the different sizes of the input data, effectively reducing the parameter count of the model. Unlike traditional convolutions that only use fixed kernel sizes, this type of operator also improves the robustness of convolution nerual nerworks, as the model can more quickly focus on discriminative features. Before executing the multi-head attention in each stage, the DEB uses a regular convolution operation to non-overlappingly divide the feature map of the original input into many patches (kernel_size, stride = patch_size). As shown in Figure 3, before and after this regular convolution operation, the DEB introduces a dynamic convolutional layer to guide the subsequent multi-head module to focus on more meaningful features:


(1) $$\begin{array}{l} H=\text{DEB}\left(X\right), \end{array}$$

Where DEB represents a single dynamic embedding block, consisting of two dynamic convolution layers and a vanilla convolution layer with a stride of 2 or 4 for downsampling. We introduce this block to integrate of all tokens before feeding them to the multi-headed self-attentive module. Such a design combining dynamic convolution has two benefits. First, the dynamic convolution operation is very friendly to varying input resolutions. Second, dynamic convolution is light-weight, which can largely alleviate the over-fitting problem encountered because of the small amount of data.

### 3.3 Parallel local-global feature extraction

Positional encoding introduces relative position relationships between multiple tokens, which is essential for ViTs to capture sequential information in the sequence. Traditionally, absolute positional encoding was first introduced into ViTs (35, 36), and this style is not friendly for different input resolutions. And relative positional encoding also does not always perform well due to its heavy computational burden (38). To improve efficiency, several recent works introducing convolutional positional encoding have been proposed to flexibly embed position relations (39, 40), and we follow them.

As previously discussed, convolution operations are effective at capturing local contextual relationships, while self-attentive mechanisms excel at capturing global dependencies. Therefore, we propose a two-branch structure that concurrently extracts local and global context features, with one branch utilizing convolution operations and the other branch calculating attention distributions. This design enables efficient and effective hierarchical representation learning of local-global context features through multi-stage stacking. Additionally, for feature alignment, we implement both branches as unified multi-head styles, i.e., local MHSA: MHSA_*l*_ and global MHSA: MHSA_*g*_,


(2) $$\begin{array}{l} V_{n}=A_{n}H_{n}, \end{array}$$

(3) $$\begin{array}{l} H^{k}=W*\text{Concat}\left[V_{1},V_{2},...,V_{n}\right]. \end{array}$$

Given a series of tokens *H*_*n*_, the token relation aggregators *A*_*n*_ capture the dependencies between them. Then, these relations are concatenated in the channel dimension, where *W* represents the learnable parameter matrix.

**Figure 4:**
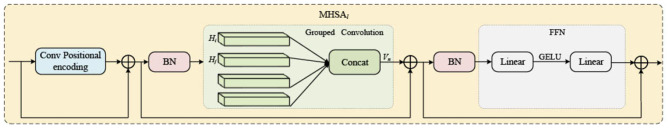
The detailed structure diagram of MHSA_*l*_. Multi-head feature extraction is achieved by grouped convolution to extract local features, followed by point convolution for feature aggregation. Similar to traditional ViTs, we add residual connections for the model to trace back to previous features. MHSA_*g*_, on the other hand, extracts and aggregates global features through fully connected layers.

*1) Local MHSA:* benefiting from the fact that convolution neural networks are specialized in focusing on local detailed features within a small region, MHSA_*l*_ is designed as a pure convolutional structure without introducing self-attention operations, as described in Figure 4. In particular, unlike the previous convolutional blocks, MHSA_*l*_ follows a transformer-like style. We extract features from various “heads” using group convolution, and aggregate multi-head features in the channel dimension. Concretely, given a series of anchor tokens {_*H*_*i*_}1:*n*_ obtained by group convolution, the MHSA_*l*_ learns the affinities between them by a local convolution operation in a small neighborhood Ω^*d* × *h* × *w*^:


(4) $$\begin{array}{l} V_{n}=W_{n}^{i-j}*\left(H_{j},H_{i}\right),\;j\in {\text{Ω}}^{d\times h\times w}, \end{array}$$

where $W_{n}^{i-j}\in {\mathbb{R}}^{d\times h\times w}$ is the learnable convolutional kernel parameter matrix, and (*i*−*j*) denotes the relative position between raw tokens *H*_*i*_ and *H*_*j*_. By doing so, each head corresponds to each channel of the feature map, enabling us to extract local discriminative features in sMRI within a limited receptive field while preserving the spatial structure of the feature map.

*2) Global MHSA:* self-attention is a natural method for capturing long-range dependencies between features and constructing discriminative global representations. In Vision Transformers (ViTs), a multi-head structure is frequently employed to process sequence information. Each “head” learns independent feature mappings and, ultimately, obtains a global view via linear aggregation:


(5) $$H_{n}=\frac{e^{Q_{n}{\left(H_{i}\right)}^{T}K_{n}(H_{j})}}{\sum_{j^{'}\in \Omega^{D\times H\times W}}e^{Q_{n}{\left(H_{i}\right)}^{T}K_{n}(H_{j'}),}}$$

where *Q*_*n*_(·) and *K*_*n*_(·) are chosen as normal fully connected transformations and the above equation is a standard *softmax* operator. Note that *j*′∈Ω^*D* × *H* × *W*^ belongs to the global, signaling the concern of the MHSA_*g*_ for global dependencies. To process the spatial dimensions of sMRI (depth *D*, height *H*, and width *W*), we convert them into a one-dimensional tensor and input it to the fully connected layer. Although traditionally this operator introduces significant computational burden, our MHSA_*g*_ is located at the post-stage of the network, where it processes feature maps that have been downsampled through multiple stages. This allows for a balance between computational efficiency and accuracy.

Local-global context features in sMRI are aggregated in multiple stages through parallel feature extraction using MHSA_*l*_ and MHSA_*g*_. Similarly to conventional ViTs, we introduce a feed-forward network (FFN) after each MHSA block. Our FFN has a specific architecture consisting of two linear layers wrapped around a nonlinear activation function (GELU). The first linear layer expands the channel dimension by the ratio of 4, while the next linear layer reduces it back to its original level. This operation facilitates the further filtering of features and improves the nonlinear expression of the model.

Considering the excessively large dimensions of the feature maps in the first two stages, it imposes a significant computational burden on the calculation of self-attention. Therefore, in the initial two stages of PlgFormer, the computation of MHSA adopts a locally based convolutional feature extraction module. Subsequently, a parallel local-global context extraction structure is employed to independently extract local features and aggregate global representations.

### 3.4 Feature fusion module

**Figure 5:**
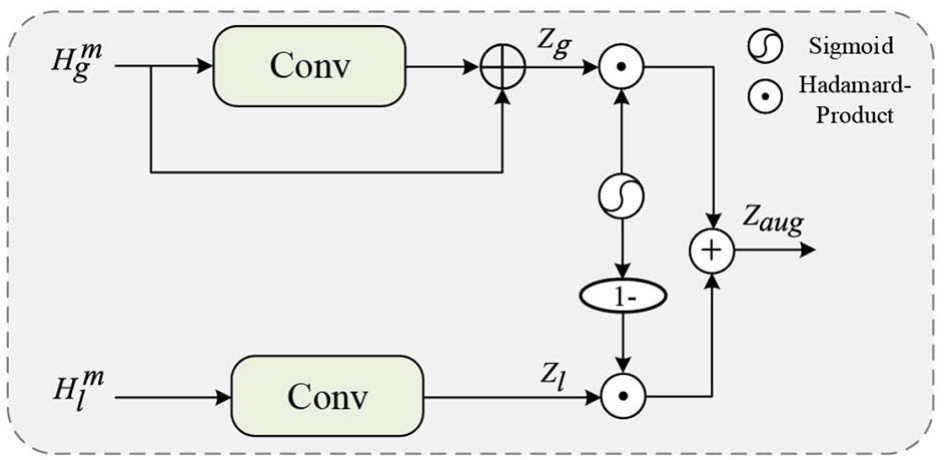
The detailed structure of the F2M.

The F2M introduces a simple gating mechanism to enable adaptive feature selection, as depicted in Figure 5. For local feature maps, a convolution with a larger kernel size of 5 × 5 × 5 is applied to focus on the more global features (the size of feature maps is 5 × 6 × 5 at this stage). Conversely, for global feature maps, point-by-point convolution is employed to preserve the global features of interest to self-attention. Finally, *Z*_*l*_ is passed through a sigmoid activation function, resulting in a mapping to values between 0 and 1 that controls the flow of local-global context features:


(6) $$\begin{array}{l} Z_{l}=W_{l}*H_{l}^{m}, \end{array}$$

(7) $$\begin{array}{l} Z_{g}=W_{g}*H_{g}^{m}+H_{g}^{m}, \end{array}$$

(8) $$\begin{array}{l} Z_{aug}=Z_{g}*\sigma \left(Z_{l}\right)+Z_{l}*\left(1-\sigma \left(Z_{l}\right)\right). \end{array}$$

It's worth noting that, in the computation of multi-head self-attention, we drew inspiration from Zhang et al. (14) and introduced residual connections for the MHSA_*g*_ module, while this design consideration was not applied to MHSA_*l*_. This choice is primarily made because residual connections assist deep networks in rapidly backpropagating shallow features. The convolutional module, having fewer network layers, does not necessitate the introduction of residual connections, as gradients can quickly propagate to the lower layers without them. However, for the globally self-attention operation based on fully connected layers, residual connections play a crucial role in facilitating better information propagation. They help alleviate the issue of gradient decay, ensuring improved preservation and dissemination of vital global information during global modeling. Here, *W*_*l*_ and *W*_*g*_ represent the learnable convolutional kernel parameter matrices, and σ(·) denoting the sigmoid activation function. The resulting feature set *Z*_*aug*_ contains both global features aggregated by self-attention and local features extracted through convolution. We obtain *Z*_*aug*_ as a one-hot tensor following global average pooling of aggregated spatial features and projection using a fully connected layer.

## 4 Experiments

In this section, we first present the two datasets used in this study in Section 4.1. Then, we present the specific experimental setup and evaluation metrics in Section 4.2. Subsequently, we introduce the comparison experiments with other existing methods in Section 4.3, and the ablation studies to validate the key components of the proposed model in Section 4.4.

### 4.1 Datasets

In this study, we employed two independent datasets to validate the performance of our proposed method, i.e., the publicly available Alzheimer's Disease Neuroimaging Initiative (ADNI) dataset and the privately available Xuanwu Neuroimaging (XWNI) dataset.

*1) ADNI dataset:* the ADNI is a large-scale, multicenter research study that aims to identify clinical, imaging, genetic, and biochemical biomarkers for the early detection and tracking of AD (41). This dataset has been extensively used in AD research, including studies on disease progression, diagnosis, and treatment. The availability of longitudinal data from multiple modalities makes it an invaluable resource for developing and evaluating machine learning algorithms for AD detection, prediction, and diagnosis. In this study, we utilized T1-weighted MRI scans from the ADNI dataset, which consists of various types of data, including clinical assessments, neuropsychological tests, MRI, PET, and genetic data. We only select data from ADNI1 dataset, and all sMRI data are acquired using a 1.5T MRI scanner. The ADNI dataset used in our study includes 1,779 samples, comprising 546 cases of NC (283 females, 263 males, 76.5 ± 5.1 years), 840 cases of mild cognitive impairment (MCI; 336 females, 504 males, 75.5 ± 7.1 years), and 393 cases of AD (198 females, 195 males, 75.3 ± 7.6 years). The demographics of participants in ADNI dataset are shown in Table 1. Moreover, we use Table 2 to show our training-testing splits number of ADNI datasets.

**Table 1:** Demographics of participants in ADNI and XWNI datasets.

			**Gender (F/M)**	**Age (years)**
Datasets	ADNI (*n* = 1,779)	CN (*n* = 546)	283/263	76.5 ± 5.1
		MCI (*n* = 840)	336/504	75.5 ± 7.1
		AD (*n* = 393)	198/195	75.3 ± 7.6
	XWNI (*n* = 711)	CN (*n* = 515)	291/222^*^	64.8 ± 24.8
		MCI (*n* = 47)	28/18^*^	62.2 ± 21.2
		AD (*n* = 149)	97/52	70.4 ± 30.6

F and M refer to female and male, respectively. Gender information is missing for 2 CN and 1 MCI subjects in the XWNI dataset and is marked with an asterisk (*).

**Table 2:** Training and testing split numbers of ADNI and XWNI datasets.

**Datasets**	**ADNI**						**XWNI**					
**Tasks**	**CN vs. AD**		**CN vs. MCI**		**CN vs. MCI vs. AD**		**CN vs. AD**		**CN vs. MCI**		**CN vs. MCI vs. AD**	
	**Train**	**Test**	**Train**	**Test**	**Train**	**Test**	**Train**	**Test**	**Train**	**Test**	**Train**	**Test**
CN	363	183	474	72	474	72	331	204	66	33	126	63
AD	330	64	-	-	330	63	100	49	-	-	100	49
MCI	-	-	486	354	486	93	-	-	30	17	30	17

*2) XWNI dataset:* the XWNI dataset was obtained from Xuanwu Hospital Capital Medical University, located in Beijing, China.^1^ This dataset includes sMRI data from patients diagnosed with AD, MCI, and NC. It stands out due to its large sample size and high-quality sMRI images. The sMRI data were acquired using a 3.0T MRI scanner and were preprocessed to improve image quality. The dataset contains both raw and preprocessed sMRI data, including skull-stripped and segmented images. A total of 711 samples were collected, including 515 cases of CN, 47 cases of MCI, and 149 cases of AD. The XWNI database is anticipated to be a valuable resource for researchers involved in AD diagnosis and related studies. The demographics of participants in XWNI dataset are shown in Table 1. Moreover, we use Table 2 to show our training-testing splits number of XWNI datasets.

**Figure 6:**
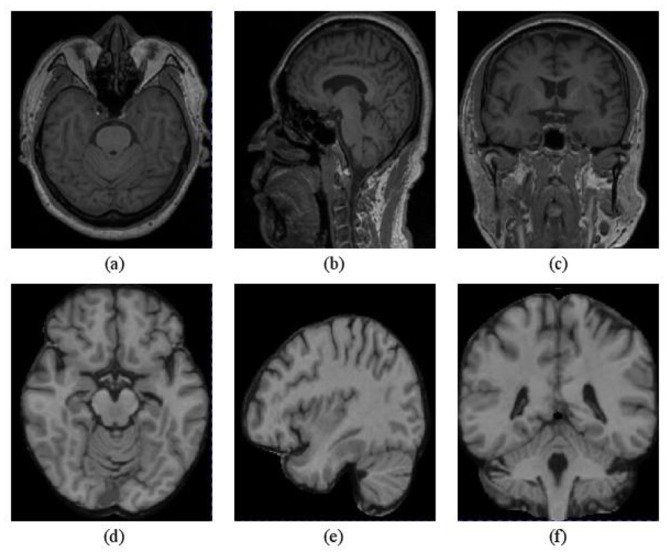
Comparison of sMRI before and after preprocessing. **(a–c)** Are raw sMRI from the axial view, the coronal view, and the sagittal view, respectively; **(d–f)** are preprocessed brain images from the corresponding view that have been cropped after matching to the MNI152_T1_1mm template.

*3) Preprocessing:* MNI152_T1 is a commonly used standard template for brain MRI image analysis and research, featuring a standard spatial coordinate system and brain structure information. Considering spatial resolution as a vital parameter affecting image quality and resolution, we initially paired the T1-weighted sMRI with the MNI152_T1_1mm template to obtain a more precise description of brain structure. Moreover, to avoid the computational burden from excessive sMRI data dimensionality, we cropped all images to *D* × *H* × *W*:148 × 192 × 156. These preprocessing methods can enhance the quality and effectiveness of sMRI data and lay the groundwork for subsequent analysis and research. Figure 6 illustrates the difference between sMRI images before and after preprocessing, indicating that the preprocessed images better capture the structural organization of brain tissue and demonstrate improved image quality.

We utilized the medical image processing tool, MONAI, to transform and augment the dataset to adapt to the model input and enhance the robustness of model training, as described in Table 3. In the table, “Augmentation” refers to the functions encapsulated in MONAI, and “Values” refers to the specific parameters corresponding to the functions.

**Table 3:** The dataset was transformed and augmented using MONAI.

**Dataset split**	**Augmentation**	**Values**
Training set	Rand spatial cropd	roi_size = [144, 176, 144]
	Histogram normalized	-
	Normalize intensityd	-
	Rand flipd	prob = 0.2 (dim:depth, heigth and width)
	Rand scale intensityd	factors = 0.1, prob = 1.0
	Rand shift intensityd	factors = 0.1, prob = 1.0
Test set	Center spatial cropd	roi_size = [144, 176, 144]
	Histogram normalized	-
	Normalize intensityd	-

### 4.2 Experimental setup and evaluation metrics

The proposed method was implemented using Python 3.7.0 and PyTorch 1.10.0. The GPU we used is one P40 with a memory of 24GB. We trained the network end-to-end using AdamW optimizer and optimize under the supervision of the cross-entropy loss function, without additional data for pre-training. PlgFormer consists of four stages, with 2, 2, 3, and 4 Multi-Head Self-Attention (MHSA) mechanisms allocated to each stage respectively. The DEB module uses 4 dynamic convolution kernels to enhance feature representation, an attention hidden ratio of 0.25 to balance performance and computational efficiency, and a temperature parameter initialized at 34 and decreased by 3 during training to progressively sharpen the attention distribution. The initial learning rate was set to 5e-4 and decayed with a cosine annealing strategy. To overcome early optimization difficulties, we performed a linear warm-up for the first 100 epochs, while the total training epochs were 400. Considering the extremely unbalanced number of samples contained in each category of the dataset, especially in the XWNI dataset, we used weighted random sampling to balance the number of samples contained in each category when loading the dataset to pursue better diagnostic performances. We conducted dichotomous (AD vs. CN and MCI vs. CN) and trichotomous (AD vs. MCI vs. CN) classifications to comprehensively evaluate the diagnostic performance of our model with varying degrees of cognitive impairment.

For model evaluation, we select a comprehensive set of metrics including accuracy (ACC), recall (REC), precision (PRE), F1-score (F1), and specificity (SPE). The calculation of each metric is as follows:


(9) $$\begin{array}{l} \text{ACC}=\frac{TP+TN}{TP+TN+FP+FN} \end{array}$$

(10) $$\begin{array}{l} \text{REC}=\frac{TP}{TP+FN} \end{array}$$

(11) $$\begin{array}{l} \text{PRE}=\frac{TP}{TP+FP} \end{array}$$

(12) $$\begin{array}{l} \text{F1}=\frac{2\times \text{REC}\times \text{PRE}}{\text{REC}+\text{PRE}} \end{array}$$

(13) $$\begin{array}{l} \text{SPE}=\frac{TN}{TN+FP} \end{array}$$

where *TP, FP, TN*, and *FN* denote true positive, false positive, true negative, and false negative, respectively. In practice, REC denotes the probability that a positive sample in the data set is correctly discriminated, and PRE denotes the proportion of all examples diagnosed as positive by the model that are in fact positive. Obviously, REC and PRE are numerically a pair of mutually exclusive indicators, so we calculate the F1-score to evaluate the performance of the model in a comprehensive way. And SPE indicates the ability of the model to determine negative samples. In addition, we also report the Area Under the Curve (AUC), which is the area under the Receiver Operating Characteristic Curve (ROC) curve, to measure the performance of the dichotomous model. is equal to 0.5, the model is equivalent to a random guess.

### 4.3 Comparison experiments

We compared our proposed method with several existing classification approaches fine-tuned on large-scale multimodal medical imaging datasets, including Med3D-ResNet-10, Med3D-ResNet-18, and Med3D-ResNet-34 (42). Additionally, we extended the original ViT model (35) to support 3D medical image classification, referred to as ViT-ori in this study. We also included methods specifically designed for Alzheimer's disease classification, covering both CNN-based and Transformer-based architectures, such as DA-MIDL (24), AMSNet (25), ResAttNet-10 (26), ResAttNet-18 (26), ViT-for-AD (43), and MCNEL (44). To comprehensively evaluate the performance of our approach, we conducted three sets of experiments on both datasets: (1) binary classification between AD and CN, (2) binary classification between MCI and CN, and (3) multi-class classification among AD, MCI, and CN subjects.

*1) Binary classification on AD and CN subjects:* accurately identifying AD plays a crucial role in clinical diagnosis as well as computer-aided diagnosis. AD is characterized by brain atrophy, gray matter atrophy, reduced brain tissue, white matter damage, enlarged sulcal gyrus, and ventricles, which can be easily detected by physicians or computers on sMRI. In this study, we employed PlgFormer to set the embedded dimension of each stage to 16, 32, 64, and 128, and each head dimension to 16. The weight decay, learning rate, and batch size were set to 5e-4, 2e-4, and 16, respectively. We conducted comparison experiments on ADNI and XWNI datasets, and the results are presented in Table 4.

**Table 4:** Quantitative comparison of our proposed PlgFormer and other existing methods for AD and CN binary classification on ADNI and XWNI datasets.

**Method**	**ACC**	**REC**	**PRE**	**F1**	**SPE**	**AUC**
**ADNI dataset**						
Med3D-ResNet-10 (42)	0.8780	0.9365	0.6941	0.7973	0.8579	0.8972
Med3D-ResNet-18 (42)	0.8943	0.9206	0.7342	0.8169	0.8852	0.9029
Med3D-ResNet-34 (42)	0.8984	0.9048	0.7500	0.8201	0.8962	0.9005
DA-MIDL (24)	0.9106	0.7619	0.8727	0.8136	0.9617	0.8618
AMSNet (25)	0.8902	0.9206	0.7250	0.8112	0.8798	0.9002
ResAttNet-10 (26)	0.9065	0.8730	0.7857	0.8271	0.9180	0.8955
ResAttNet-18 (26)	0.8821	0.7302	0.7931	0.7603	0.9344	0.8323
ViT-ori (35)	0.6870	0.8095	0.4397	0.5698	0.6448	0.7272
ViT-for-AD (43)	0.9000	**1.0000**	0.8750	**0.9333**	0.6667	0.8335
MCNEL (44)	0.8998	0.8750	0.8750	0.8750	0.9167	0.8958
**PlgFormer (ours)**	**0.9431**	0.8730	**0.9016**	0.8871	**0.9672**	**0.9201**
**XWNI dataset**						
Med3D-ResNet-10 (42)	0.8933	0.8367	0.6833	0.7523	0.9069	0.8769
Med3D-ResNet-18 (42)	0.9012	0.8163	0.7143	0.7872	0.9216	0.8718
Med3D-ResNet-34 (42)	0.8814	0.8367	0.6508	0.7321	0.8922	0.8648
DA-MIDL (24)	0.9091	0.7347	0.7826	0.7579	**0.9510**	0.8428
AMSNet (25)	0.9209	0.8163	0.7843	0.8000	0.9461	0.8812
ResAttNet-10 (26)	0.9091	0.7959	0.7501	0.7723	0.9363	0.8781
ResAttNet-18 (26)	0.9130	0.8776	0.7288	0.7963	0.9216	0.8996
ViT-ori (35)	0.8735	0.6226	0.6889	0.6596	0.9314	0.7820
ViT-for-AD (43)	0.8824	0.9000	**0.9000**	0.9333	0.9167	0.8958
MCNEL (44)	0.9105	0.9050	0.8700	**0.9400**	0.9300	0.9100
**PlgFormer (ours)**	**0.9407**	**0.9184**	0.8036	0.8517	0.9461	**0.9322**

The bold values indicate the best value in the current column.

As elaborated in Table 4, our proposed PlgFormer achieves superior performance on ADNI and XWNI datasets in most cases. PlgFormer outperforms other methods in most of the evaluation metrics on both datasets, with ACC = 0.9431, PRE = 0.9016, F1 = 0.8871, SPE = 0.9672, and AUC = 0.9201 on ADNI dataset and ACC = 0.9407, REC = 0.9184, PRE = 0.8036, F1 = 0.8517, and AUC = 0.9021 on XWNI dataset. Although PlgFormer did not achieve the best performance in some metrics, such as REC on ADNI dataset and SPE on XWNI dataset, it notably outperformed other existing methods on several metrics. Notably, PlgFormer achieved the highest accuracy, suggesting its excellent performance in diagnosing AD patients. These results demonstrate the effectiveness and potential of using PlgFormer for binary classification in AD and CN, which can aid in early diagnosis and intervention for AD.

*2) Binary classification on MCI and CN subjects:* identifying individuals with MCI is a crucial task for early intervention in AD, especially since there are currently no well-established treatment options for AD. However, using sMRI alone to perform this task is challenging, as the brain regions of patients with MCI usually do not undergo significant morphological changes. In this study, we set the embedding dimension of each stage of PlgFormer to 16, 32, 64, 96, while keeping the other hyperparameters the same as those for the AD and CN binary classification. The results of a detailed comparison with other existing methods are presented in Table 5.

**Table 5:** Quantitative comparison of our proposed PlgFormer and other existing methods for MCI and CN binary classification on ADNI and XWNI datasets.

**Method**	**ACC**	**REC**	**PRE**	**F1**	**SPE**	**AUC**
**ADNI dataset**						
Med3D-ResNet-10 (42)	0.7160	0.7260	0.9146	0.8094	0.6667	0.6963
Med3D-ResNet-18 (42)	0.7958	0.8192	0.9265	0.8696	0.6806	0.7499
Med3D-ResNet-34 (42)	0.7981	**0.8531**	0.8978	0.8751	0.5278	0.6904
DA-MIDL (24)	0.7887	0.8192	0.9177	0.8657	**0.8194**	0.7077
AMSNet (25)	0.7840	0.8362	0.8970	0.8655	0.5278	0.6820
ResAttNet-10 (26)	0.7864	0.8305	0.9046	0.8660	0.5694	0.7000
ResAttNet-18 (26)	0.7934	0.8390	0.9055	0.8710	0.5694	0.7042
ViT-for-AD (43)	0.7959	0.6250	0.7143	0.6667	0.8788	0.7368
MCNEL (44)	0.8000	0.7500	0.7500	0.7500	0.8333	0.7107
**PlgFormer (ours)**	**0.8216**	0.8503	**0.9290**	**0.8879**	0.6806	**0.7654**
**XWNI dataset**						
Med3D-ResNet-10 (42)	0.7800	0.8824	0.6250	0.7317	0.7273	0.7796
Med3D-ResNet-18 (42)	0.8000	0.7647	0.6842	0.7222	0.8182	0.7832
Med3D-ResNet-34 (42)	0.8200	0.7059	0.7500	0.7273	0.8788	0.7905
DA-MIDL (24)	0.8000	0.7059	0.7059	0.7059	0.8485	0.7745
AMSNet (25)	0.8000	0.8235	0.6667	0.7368	0.7879	0.8057
ResAttNet-10 (26)	0.8000	0.8824	0.6522	0.7500	0.7576	0.8200
ResAttNet-18 (26)	0.7800	**0.9412**	0.6154	0.7442	0.6970	0.8191
ViT-for-AD (43)	0.8235	0.9000	**0.8182**	0.8571	0.7143	0.8285
MCNEL (44)	0.8367	0.6875	0.7851	0.7333	**0.9090**	0.8421
**PlgFormer (ours)**	**0.8600**	0.8235	0.7778	**0.8000**	0.8788	**0.8512**

The bold values indicate the best value in the current column.

The results presented in Table 5 demonstrate that our proposed PlgFormer outperforms other existing methods on both ADNI and XWNI datasets, with the highest metrics achieved in most cases. For example, on the ADNI dataset, our method achieved a PRE of 0.9290 and an AUC of 0.7654, while on the XWNI dataset, our method achieved a PRE of 0.7778 and an AUC of 0.8512. It is worth noting that MCI patients exhibit less significant structural alterations in brain regions on sMRI compared to AD patients. Consequently, the binary performance of the MCI and CN subjects was not as good as that of the AD and CN subjects on both data sets. As illustrated in Table 5, our PlgFormer achieves notable performance in F1 scores on both datasets, thus demonstrating its ability to distinguish between MCI and CN subjects.

*3) Triple classification on AD, MCI, and CN subjects:* performing a triple classification experiment using sMRI on AD, MCI, and CN subjects is of great significance for computer-aided AD diagnosis. Currently, there is no effective cure for AD, underscoring the importance of early definitive detection to enable early intervention and treatment. MCI, as a transitional stage between normal aging and dementia, significantly increases the risk of developing AD. Therefore, accurately diagnosing and differentiating individuals

with MCI and AD from those with normal cognitive function (CN) is crucial to facilitate the early detection and management of AD.

Table 6 presents quantitative comparisons, revealing that our proposed PlgFormer outperforms other existing methods, particularly in the recognition of AD subjects, notably in the ADNI dataset. We attribute this to the flexibility of PlgFormer in addressing both local and global requirements, making it more sensitive to significant structural changes in brain regions. In general, our method demonstrated remarkable performance on most metrics, providing compelling evidence of its potential use in clinical practice for computer-aided diagnosis. Furthermore, our review of existing studies utilizing sMRI for AD diagnosis reveals that limited attention has been given to the three-way classification of AD, MCI, and CN. We acknowledge the inherent difficulty of this task, as current models often struggle to capture the subtle and discriminative features present in sMRI. Nevertheless, given its substantial clinical relevance, we strongly encourage future research to further explore and address this challenging problem.

**Table 6:** Quantitative comparison of our proposed PlgFormer and other existing methods for AD MCI and CN triple classification on ADNI and XWNI datasets.

**Method**		**AD**				**MCI**			
	**ACC**	**REC**	**PRE**	**F1**	**SPE**	**REC**	**PRE**	**F1**	**SPE**
**ADNI dataset**									
Med3D-ResNet-10(42)	0.5864	0.4000	0.2424	0.3019	0.8111	0.6036	0.7913	0.6848	0.5726
Med3D-ResNet-18 (42)	0.5908	0.3000	0.2222	0.2553	0.8413	0.6126	0.7969	0.6927	0.5806
Med3D-ResNet-34 (42)	0.5996	0.4333	0.3377	0.3796	0.8715	0.6006	**0.8197**	0.6932	0.6452
DA-MIDL (24)	0.6053	0.3968	0.2778	0.3268	0.8474	**0.6582**	0.7664	**0.7082**	0.4741
AMSNet (25)	0.6012	0.4603	0.2929	0.3580	0.8357	0.6384	0.7740	0.6997	0.5111
ResAttNet-10 (26)	0.5667	0.3167	0.2879	0.3016	**0.8816**	0.6036	0.7614	0.6734	0.4919
ResAttNet-18 (26)	0.5886	0.2833	0.2537	0.2677	0.8741	0.6366	0.7823	0.7020	0.5242
ViT-for-AD (43)	0.5882	0.4000	0.4444	0.4211	0.8750	0.6364	0.7000	0.6667	0.4286
MCNEL (44)	0.6105	0.5800	0.4500	0.5100	0.8600	0.6400	0.7300	0.6850	0.5800
**PlgFormer (ours)**	**0.6228**	**0.7619**	**0.5161**	**0.6154**	0.7273	0.3871	0.6545	0.4865	**0.8593**
**XWNI dataset**									
3D-ResNet-10 (42)	0.8047	0.7347	0.8372	0.7826	0.9114	0.4706	0.6154	0.5333	0.9550
3D-ResNet-18 (42)	0.8125	0.8367	0.8913	0.8632	0.9367	**0.6471**	0.4231	0.5116	0.8649
3D-ResNet-34 (42)	0.8203	0.7143	0.8750	0.7865	0.9367	0.5882	0.7143	0.6452	0.9640
DA-MIDL (24)	0.8438	0.7755	0.8636	0.8172	0.9241	0.5882	0.7143	0.6452	0.9640
AMSNet (25)	0.8281	0.7551	0.8605	0.8043	0.9241	0.5294	0.6000	0.5625	0.9459
ResAttNet-10 (26)	0.8047	0.7755	0.8261	0.8000	0.8987	0.5882	0.6250	0.6061	0.9459
ResAttNet-18 (26)	0.8281	0.7551	0.8605	0.8043	0.9241	0.5294	0.6923	0.6000	0.9640
ViT-for-AD (43)	0.8359	0.7857	0.8696	0.8020	0.9300	0.5588	0.6667	0.6061	0.9550
MCNEL (44)	0.8500	0.8250	0.8800	0.7700	0.9350	0.6200	0.8000	**0.6850**	0.9700
**PlgFormer (ours)**	**0.8672**	**0.8571**	**0.9130**	**0.8842**	**0.9494**	0.5833	**1.0000**	0.5833	**1.0000**

The bold values indicate the best value in the current column.

### 4.4 Ablation studies

In this subsection, we conducted ablation studies on AD and CN binary classification on ADNI dataset to evaluate the impact of various key components in PlgFormer on model representation capacity. Similarly, we selected ACC, REC, PRE, F1, SPE, and AUC as evaluation metrics. The corresponding results are presented in Table 7, *L* and *G* respectively represent MHSA_*l*_ and MHSA_*g*_, ✓denotes leaving the corresponding component in place, whereas × denotes replacing it with another multi-head self-attention module.

**Table 7:** Ablation studies on individual components of the proposed PlgFormer.

**Method**	**F2M**	**DEB**	***L***	***G***	**ACC**	**REC**	**PRE**	**F1**	**SPE**	**AUC**
Med3D-ResNet-34 (42)	-	-	-	-	0.8984	0.9048	0.7500	0.8201	0.8962	0.9005
DA-MIDL (24)	-	-	-	-	0.9106	0.7619	0.8727	0.8136	0.9617	0.8618
ResAttNet-10 (26)	-	-	-	-	0.9065	0.8730	0.7857	0.8271	0.9180	0.8955
PlgFormer	×	✓	✓	✓	0.8821	0.8571	0.7297	0.7883	0.8907	0.8739
PlgFormer	✓	×	✓	✓	0.9146	0.7778	0.8750	0.8235	0.9617	0.8698
PlgFormer	✓	✓	×	✓	0.7480	**1.0000**	0.5040	0.6702	0.6612	0.8306
PlgFormer	✓	✓	✓	×	0.8984	0.8413	0.7794	0.8092	0.9180	0.8797
PlgFormer	✓	✓	✓	✓	**0.9431**	0.8730	**0.9016**	**0.8871**	**0.9672**	**0.9201**

✓Denotes leaving the corresponding component in place whereas × denotes removing it. All experiments are conducted on ADNI dataset to distinguish AD and CN subjects. The bold values indicate the best value in the current column.

According to the results presented in Table 7, it is evident that convolutional operations play a crucial role in the PlgFormer model we designed. When MHSA_*l*_ was replaced with MHSA_*g*_, the performance of the model dropped significantly (ACC decreased from 0.9431 to 0.7480). We attribute this phenomenon to the small sample size of our dataset, as pure self-attention modules require a large amount of data to demonstrate their effectiveness. We also observed a slight drop in performance when MHSA_*g*_ was replaced with a local MHSA_*l*_, suggesting that global features are still necessary for AD classification using sMRI. This conclusion was supported by the fact that attention modules were embedded in the DA-MIDL and ResAttNet-10 architectures. Furthermore, when we removed the designed DEB, the performance slightly decreased, indicating that encoding image patch sequences dynamically is a meaningful operation. In addition, F2M is also an important and effective module for feature fusion, as replacing it with a simple concatenation caused a decrease in all evaluation metrics.

We evaluated the effects of dynamic convolution on overfitting and generalization, as shown in Table 8. The results indicate that when dynamic convolution is used, the model demonstrates similar training errors but reduces validation errors, highlighting its capability to mitigate overfitting. Furthermore, dynamic convolution leads to lower errors in the testing set of the XWNI dataset, suggesting improved generalization on other datasets. It is worth noting that we conducted experiments across 5 independent runs with different random seeds and reported the mean and standard deviation of the final validation loss, in order to evaluate the training stability of DEB. The results show that the DEB-enhanced model achieves a lower average loss with reduced variance, demonstrating improved robustness and stability.

**Table 8:** Ablation studies of DEB.

**Method**	**DEB**	**loss_train**	**loss_val**
PlgFormer	×	0.0573 ± 0.0137	0.7807 ± 0.0894
PlgFormer	✓	0.0611 ± 0.0088	0.4760 ± 0.0836

All experiments are conducted on ADNI dataset to distinguish AD and CN subjects. loss_train is the loss value in the training data, loss_val represents the loss value in the validation data.

### 4.5 Visualization

**Figure 7:**
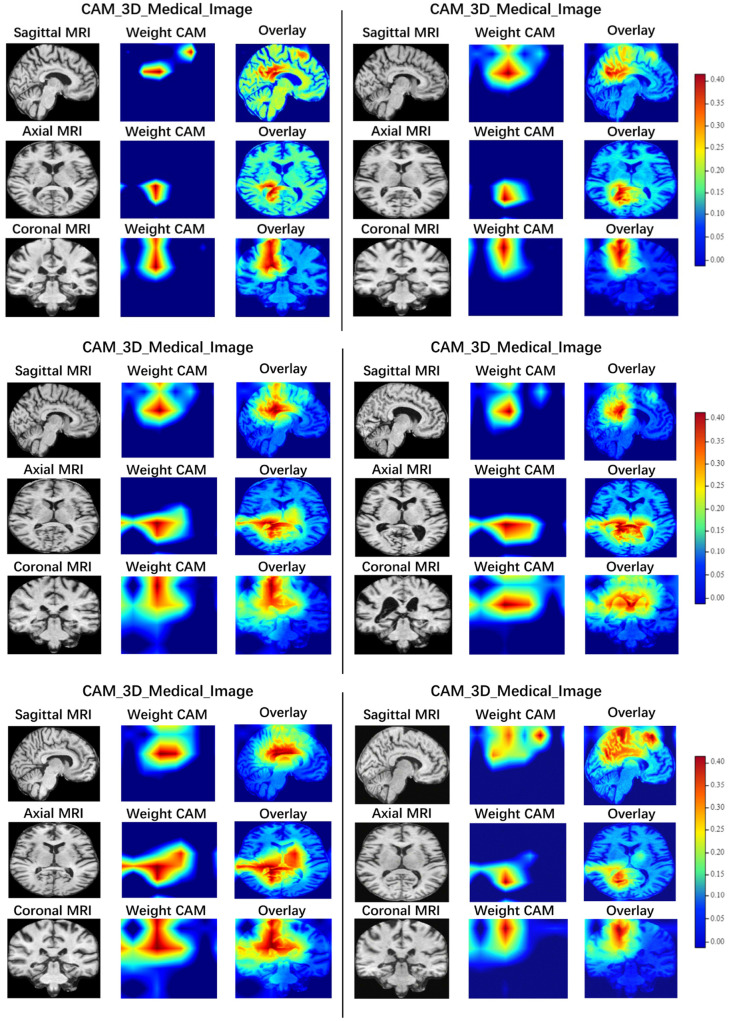
Salient maps of sagittal, axial and coronal sMRI slices generated by Grad-CAM. The visualization results allow to observe the features of network interest.

To provide human physicians with reliable and accurate computer-aided diagnostic results, we employed Grad-CAM (45) to generate sMRI slice heat maps in sagittal, axial, and coronal planes, as illustrated in Figure 7. To produce high-resolution heat maps that are easy to interpret, we applied 3D Grad-CAM at a lower layer with a resolution of 36 × 44 × 36 (*D* × *H* × *W*). In the process of visualization, the reshaped tensor of dimensions 36 × 44 × 36 was restored to its original resolution to facilitate a more detailed examination of features attended to by the neural network. The Grad-CAM procedure commenced with the loading of a pre-trained model alongside the original sMRI input. Subsequently, an intermediate layer's output and its gradients with respect to the output were selectively chosen. The visual representation of the feature maps derived from this output was then obtained. Following this, the impact of the gradients on the output of the target layer was quantified, yielding weight factors indicative of the significance of the feature maps. These weights were employed to project the importance of the feature maps back onto the input image. Finally, the application of these weights to the target layer's output resulted in the generation of a heatmap. This heatmap provides insights into which regions of the input image hold critical information for the model's predictive capacity.

We have drawn GradCAM using six patients with AD, as illustrated in Figure 7, all of whom are from the test set of the ADNI dataset. Additionally, We have analyzed the visualization results for these patients using the ICBM 152 template. First, we aligned the sMRI data of AD patients with the template, and then analyzed the brain regions corresponding to the areas of interest (high-luminance areas) of our model. After analysis, we found that the brain regions that our model focuses on include: Posterior Cingulate, Precuneus, Isthmus Cingulate, and Lateral Ventricle. We have consulted with doctors who diagnose AD clinically, and found that the Posterior Cingulate and Precuneus regions are consistent with the areas that doctors focus on during clinical diagnosis. This further confirms that the features extracted by our method are not only meaningful for deep neural network models but also provide credible diagnoses of AD via sMRI for human physicians (46).

Moreover, the visualization results obtained using Grad-CAM empower physicians to better understand and interpret the classification decision of our model. By providing a visual reference, physicians can easily validate the reasoning behind a diagnosis and identify any potential shortcomings or biases in the model. This serves as a valuable tool for improving the interpretability and transparency of Computer-aided diagnosis (CAD), and ultimately helps build trust between physicians and machine learning models.

## 5 Conclusions

Using sMRI for computer-aided diagnosis is significant for early detection and timely intervention of AD. In this paper, we propose PlgFormer, a unified and parallel approach that combines CNNs and pure self-attention mechanisms to extract local-global context features in sMRI with discriminative value for AD diagnosis. Our designed DEB introduces dynamic convolutions that adaptively adjust the kernel size based on the input size, while our designed F2M adaptively fuses the extracted local and global features through a gating mechanism. On publicly available ADNI and privately held XWNI datasets, our PlgFormer achieved state-of-the-art performance compared to existing methods in AD vs. CN binary classification, MCI vs. CN binary classification, and AD vs. MCI vs. CN triple classification tasks. Saliency maps generated by Grad-CAM confirmed that our proposed method can help human experts identify lesions quickly in sMRI. Further studies could investigate the potential of PlgFormer on other datasets and explore its application in other areas of medical image analysis. Our research anticipates practical clinical applications.

## Data Availability

The datasets presented in this article are not readily available because the XWNI dataset used in this study is not publicly available due to patient privacy concerns and institutional data sharing restrictions. Access to the dataset is limited by the policies of Xuanwu Hospital Capital Medical University and requires specific institutional approvals. Requests to access the datasets should be directed to Zhixiong Li, 865818683@qq.com.

## Footnotes

^1^This study was performed in line with the principles of the Declaration of Helsinki. Ethical Approval was granted by the Ethics Committee of Xuanwu Hospital Capital Medical University (No. 2017046). All subjects signed informed consent forms.
